# Crossroads of Neurodermatology: Trigeminal Trophic Syndrome

**DOI:** 10.7759/cureus.51760

**Published:** 2024-01-06

**Authors:** Dimitra Xenopoulou, Evelyn Greco

**Affiliations:** 1 Dermatology, New York Institute of Technology College of Osteopathic Medicine, Old Westbury, USA

**Keywords:** nonhealing ulcer, neurodermatology, trigeminal neuralgia, cutaneous paresthesia, trigeminal trophic syndrome

## Abstract

A 98-year-old female, with a past medical history of trigeminal neuralgia (TN) and non-melanoma skin cancer, presented with a crescent-shaped ulcer on her right nasal ala that had been present for months. On exam, the patient was aware of her issue, readily admitted to manipulation of the area, and had a past medical history significant for TN. The patient's history and clinical presentation led to a diagnosis of trigeminal trophic syndrome (TTS). TTS is an extremely rare, ulcerative condition that can arise in patients suffering from TN. While TN itself is well-documented, treatment is often challenging and usually focused on achieving symptomatic relief; for this patient, she did not achieve adequate management of her neuropathic symptoms, and her condition progressed to TTS. Thus, given the patient’s ongoing multi-modal TN treatment, she was encouraged not to pick or manipulate the area to the best of her ability to curb the extent of ulceration. Given that TTS is so infrequently seen, we are hopeful that, by identifying the specifics of the underlying neuronal aberrancies in the future, we may be able to better grasp TTS’s pathophysiology, ulcer development, and potential future treatment options.

## Introduction

Trigeminal neuralgia (TN) is a neurological condition that presents with intense, intermittent, and unpredictable pain in one or more trigeminal nerve (CN V) branches. Pain can last anywhere from seconds to minutes and range from a few to several hundred attacks daily. Although the exact incidence of TN is unknown, female adults over the age of 50 are most commonly affected [1].

Trigeminal trophic syndrome (TTS) is a rare, ulcerative condition that can arise in patients suffering from TN. TTS can arise secondary to damage to CN V and/or its central sensory connections, often in patients treated for TN. Initially documented as a cutaneous ulceration in the trigeminal dermatome by German internist and neurologist Adolf Wallenberg in 1901, there have been less than 200 reports of this condition in the literature since its first description [2].

This exceedingly rare form of cutaneous dysesthesia usually presents with anesthesia, paresthesia, and unilateral ulceration of the lateral nasal ala with pathognomonic sparing of the nasal tip, since that area is supplied by the medial branch of the anterior ethmoidal nerve and not CN V [3].

Although the precise mechanisms for ulcer development in TTS have not been elucidated at this time, it is thought that self-manipulation with rubbing and/or scratching of the dysesthetic regions appears to be a contributing factor and can lead to erosion of the nasal ala [4].

## Case presentation

**Figure 1:**
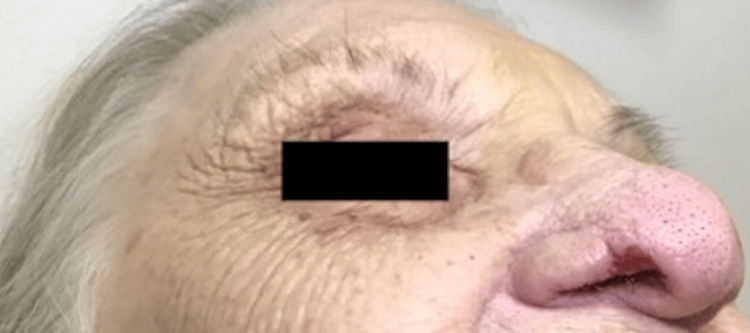
Lateral view of R nasal ala affected with trigeminal trophic syndrome (TTS)

**Figure 2:**
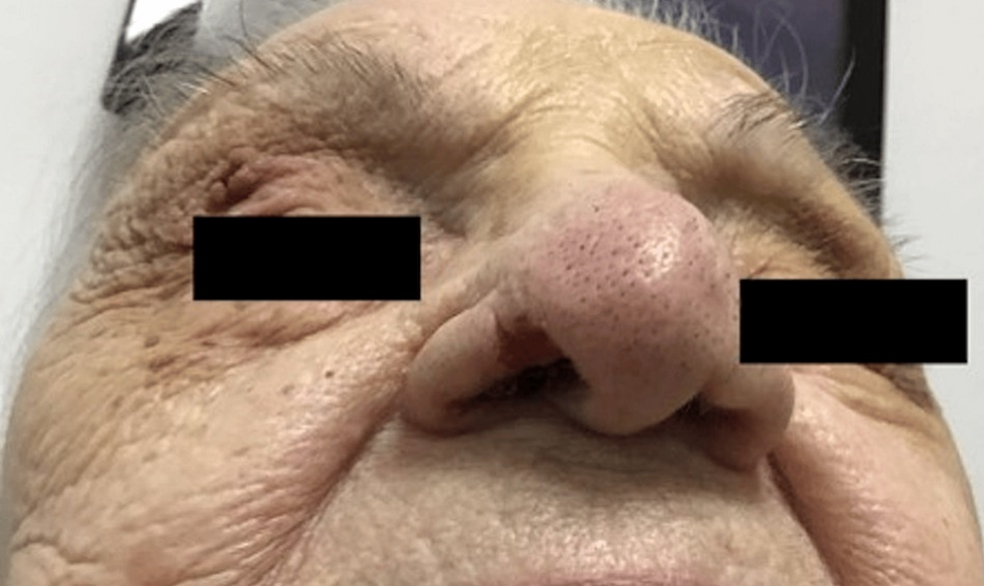
Anterior view of R nasal ala affected with trigeminal trophic syndrome (TTS)

This case report centers on a 98-year-old female, with a significant past medical history of TN who presented with a crescent-shaped ulcer on her right nasal ala that had been present for months (Figures 1, 2). The etiology for and duration of her TN was not elicited during the patient's visit. However, she was aware of her nasal ulceration and readily admitted to manipulation of the area.

Cutaneous malignancies and infections are included in the differential for TTS [5], but the latter has no definitive features on histology and only shows chronic ulceration with minimal inflammatory infiltrate. Thus, the decision to forego biopsy in order to rule out the malignancy and/or infection was made given the patient’s age and characteristic presentation.

Dermatitis artefacta could also be considered as a possible diagnosis due to the self-manipulation [6], but the patient's history of TN and clinical presentation led to a diagnosis of TTS.

Lastly, studies to allocate the site of nerve injury, such as magnetic resonance imaging (MRI), were considered, but the decision was made to forego this intervention again due to the patient’s age and characteristic presentation.

While TN itself is well-documented, treatment is often challenging and usually focused on achieving symptomatic relief [7]; in our case, the patient’s TN was treated with lidocaine 5% adhesive patches, carbamazepine 200 mg, and gabapentin 600 mg, along with various over-the-counter medications for curbing her associated ophthalmic and nasal symptoms. Despite the multi-faceted approach to treating her TN, the patient did not achieve adequate management of her neuropathic symptoms, and she developed TTS. Our clinical recommendation was that she avoid further manipulation of the area, and no other additional intervention was implemented.

## Discussion

TTS is a rare cutaneous dysaesthesia that can arise when a branch of the CN V is damaged. Patients experience both anesthesia and paraesthesia due to the aforementioned nerve damage, which leads to the self-manipulation of the area in order to achieve symptomatic relief [4]. The diagnosis of TTS is made clinically with the aforementioned presentation; biopsy of the affected area is avoided in these patients since findings are non-specific, often showing inflammatory and/or fibrosing processes that are not unique to this condition [8].

There is currently no consensus on the management and treatment of TTS; thus, the approach is often a multi-disciplinary one, which can involve multiple specialties. Patients are encouraged to perform behavioral modifications, and they are given pharmacological interventions, such as anti-convulsants, and potentially may even undergo surgery [9].

Future investigation may be done into the discrimination of tactition, thermoception, and/or nociception in TTS patients so as to better understand the specific somatosensory deficits that exist with CN V, especially since it is a nerve responsible for a bulk of varied information. We are hopeful that, by identifying the specifics of the underlying neuronal aberrancies, we may be able to better grasp this condition’s pathophysiology, ulcer development, and potential future treatment options.

## Conclusions

Due to the fact that TTS is difficult to identify and treat, it is imperative that we detail and monitor any occurrences that we may come across in our clinical practice, in hopes of strengthening our understanding and recognition of this syndrome. Furthermore, we believe that early identification of TN can potentially prevent the worsening of ulceration, preventing possible infection, and curbing future complications.
